# Giant lipoblastoma of the buttock

**DOI:** 10.11604/pamj.2019.33.63.17991

**Published:** 2019-05-28

**Authors:** Zied Jlalia, Dhia Kaffel

**Affiliations:** 1 Pediatric Orthopedics Department, Kassab Institute of Orthopedic Surgery, Ksar Said, Tunisia; 2 Rheumatology Department, Kassab Institute of Orthopedic Surgery, Ksar Said, Tunisia

**Keywords:** Tumors soft tissues, lipoblastoma, children

## Image in medicine

Ten years old girl was referred to our consultation for a tumefaction of the right buttock, it was soft consistency and mobile in relation to the deep planes (A). Magnetic resonance imaging reveals a large fatty lobulated mass well encapsulated with septa with low signal T1/T2 (B). Pathological examination of the resection piece confirmed the diagnosis of lipoblastoma (C). Lipoblastoma is a rare benign pediatric tumor derived from embryonic fat. It is often morphologically indistinguishable from primitive myxoid mesenchymal tumor of infancy. Translocations affecting the 8q11-13 region are commonly reported with lipoblastoma and proper diagnosis requires cytogenetic analysis to distinguish it from malignant myxoid liposarcoma. It is important to consider lipoblastoma in the diagnosis of a rapidly enlarging fatty mass in children. Complete resection is the only definitive treatment and should not be delayed when impingement on surrounding structures is imminent.

**Figure 1:**
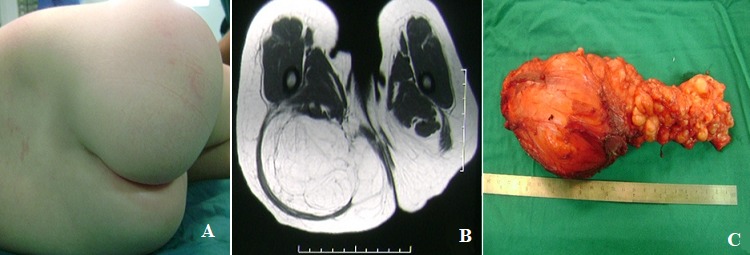
A) tumefaction of the right buttock, it was soft consistency and mobile in relation to the deep planes; B) pelvic MRI showed a large fatty lobulated mass well encapsulated with septa, with low signal T1/T2; C) pathological examination of the resection piece confirmed the diagnosis of lipoblastoma

